# Evaluation of Sediments from the River Drava and Their Potential for Further Use in the Building Sector

**DOI:** 10.3390/ma15124303

**Published:** 2022-06-17

**Authors:** Vilma Ducman, Karmen Fifer Bizjak, Barbara Likar, Mitja Kolar, Ana Robba, Jernej Imperl, Mojca Božič, Boštjan Gregorc

**Affiliations:** 1 Slovenian National Building and Civil Engineering Institute, Dimičeva ulica 12, 1000 Ljubljana, Slovenia; karmen.fifer@zag.si (K.F.B.); barbara.likar@zag.si (B.L.); 2 Faculty of Chemistry and Chemical Technology, University of Ljubljana, Večna Pot 113, 1000 Ljubljana, Slovenia; mitja.kolar@fkkt.uni-lj.si (M.K.); ana.robba@fkkt.uni-lj.si (A.R.); jernej.imperl@fkkt.uni-lj.si (J.I.); 3 Hydropower Plants, Dravske Elektrarne Maribor d. o. o., Obrežna Ulica 170, 2000 Maribor, Slovenia; mojca.bozic@dem.si (M.B.); bostjan.gregorc@dem.si (B.G.)

**Keywords:** river sediments, pollution, heavy metals, clay industry, levees

## Abstract

Sedimentation is a naturally occurring process of allowing particles in water bodies to settle out of the suspension under a gravity effect. In this study, the sediments of the Drava River were fully investigated to determine the heavy metal concentrations along the river and their potential reuse in the construction sector. Naturally dehydrated sediments from the Drava River were tested as an additive for the production of fired bricks. The dredged sediments were used as a substitute for natural brick clay in amounts up to 50% by weight, and it was confirmed that up to 20% by weight of the added sediment could be used directly in the process without critically affecting performance. Finally, the naturally dehydrated sediments were also evaluated for their use as a filling material in the construction of levees. The natural moisture content of the dehydrated sediment was too high for it to be used without additives, so quicklime was added as an inorganic binder. The test results showed an improvement in the geotechnical properties of the material to such an extent that it is suitable as a filling material for levees.

## 1. Introduction

The hydropower plant Dravske elektrarne Maribor (DEM), Slovenia’s largest producer of electricity from renewable energy sources, operates eight hydropower plants on the Drava River. Sediment management presents one of the maintenance measures implemented by DEM on the storage reservoirs on the Drava River. With appropriate maintenance work, they ensure the optimum operation of the hydropower plants and prevent the harmful function of the water. Water reservoirs can accumulate between 30,000 and 40,000 m^3^ of sediment each year. The sediment is currently treated with conventional management solutions, i.e., the disposal of the sludge in landfills or the reintegration of the sediment into rivers and aquatic ecosystems. With insufficient space for sludge disposal in the Drava valley and new regulations in place, new sediment-management solutions are needed [1].

For many years, dredged sediments have been considered waste and have been mainly landfilled or reintegrated into rivers and aquatic ecosystems. When dredged material, i.e., sediments, are stored or selected for land management, they have to be registered in the European List of Waste. This study explores new solutions for the use of sediments as a natural and renewable resource for the production of new value-added products, so that sediments can be converted from waste into a renewable production source, e.g., for the production of bricks.

Sediment pollution with heavy metals is an important environmental problem all over the world, especially in mining regions [2,3,4]. Numerous mines and smelters, such as Bleiberg-Kreuth in Austria, Cave del Predil in Italy, and Mežica in Slovenia, have had a significant impact on the chemical composition of the Meža and Drava River sediments and present today’s sediment state [5]. After the mines, the smelters, and the processing facilities were closed in 1995, the spoil heaps containing low-grade ore and ore-processing wastes persisted as a source of potentially toxic elements in the environment [5]. Various publications of heavy metal pollution of the Meža River sediments have shown very high contents of Pb, Zn, and Cd in sediments [6]. The contents of heavy metals vary but generally do not decrease with time, which indicates that mine and ore processing waste deposits represent a steady inflow of toxic elements and thus contribute considerably to the total metal load in the Meža and Drava Rivers [7].

The mineralogy of Zn and Pb host phases in the Mežica mining environment has been also extensively studied. The complete characterization of the metal-bound mineral phases and the metal loadings was presented by [6,7]. The results showed that Pb- and Zn-bearing ore minerals, such as cerussite (PbCO_3_), sphalerite (ZnS), and smithsonite (ZnCO_3_), are the prevailing metal-bearing phases in river sediments in the Meža River and can also be found in the Drava River. According to the reported solubility constants, all the mentioned minerals are stable, i.e., sphalerite (ZnS) with log Ksp = −24 [8], appearing quite stable, cerussite (PbCO_3_) with log Ksp = −13.1 [9], and smithsonite (ZnCO_3_) with log Ksp = −10 [10], also ensuring good immobilization of Pb and Zn but more pH-dependent behavior. Their good immobilization is an excellent basis for the use of sediments in construction products as the leaching of heavy metals is difficult even in the sediments themselves. For successful separation of heavy metal from minerals, a highly acidic environment is needed in the presence of oxidizing agents.

There are many methods for heavy-metal-contaminated sediment remediation; however, these methods depend on sediment and metal characteristics (e.g., grain size fractions, and physical and chemical properties). The key to successful sediment washing lies in the selection of washing agent. Up till now, a variety of washing agents (acids, surfactants, redox agents, and chelating agents) have shown effectiveness in the remediation of heavy-metal-contaminated soils and sediments. However, many of them also have drawbacks (such as high price and disrupting soil structure). Current treatment methods aimed at restoring contaminated sediments to their normal state are similar to those used for soil and include physical (centrifugation, flocculation, hydrocyclones, sieving, sedimentation, etc.), chemical (chemical leaching, chemical oxidation, chemical fixation, electrokinetic remediation, etc.) [11], and biological methods [12]. Although much is known about the technologies used to remediate soils contaminated with heavy metals [13], much less is known about the treatment of sediments [14,15,16,17]. The characteristics of sediments can differ significantly from those of soils [18], and technologies that work for soils may therefore not be as efficient for sediments. Taking into account the presence of heavy metals, we planned to prepare sediment-based building products in a way that they will not leach during their use.

The clay-based industry is constantly looking for possible additives to clay mixtures. Such additives can replace virgin clay and save resources or positively influence the production process or product itself. The mineralogical composition of sediments as a potential substitute for brick making is important because the processes during formation, drying, and firing depend on it. It is known from experience and literature that the content of clay particles in brick clay can range from 20 to 60%; in practice, it is usually 30%. Quartz affects the product properties, the drying process (decreases the sensitivity), and the firing. The phase transition of quartz at 573 °C during the cooling phase should be taken into account as it is associated with volumetric changes and can lead to cracks in the products if the cooling process is not properly controlled. The carbonate content, if finely dispersed, can be as high as 20–25%; only lime inclusions larger than 1 mm can pose a problem [19,20]. A number of publications [20,21,22,23,24,25] on the use of different sediments in the brick sector have already been carried out, and the feasibility has been confirmed, but still each individual sediment needs to be tested in combination with the clay. Bricks with 50 wt% sediment from the Bremen harbor were produced on an industrial-scale [21]. The tests performed on the bricks did not reveal leaching of heavy metals to an extent that would have a hazardous impact on the environment (soil, groundwater) and limit the use of the dredged sediment. During the sintering process, most metal contaminants were bound into new minerals and immobilized within the brick matrix [26]. Samara et al. produced bricks with 15% wt% contaminated sediment from the Dampremy–Charleroi region of Belgium as a substitute for quartz sand [23]. The substitution resulted in a significant increase in the compressive strength and firing shrinkage of the bricks, as well as a decrease in porosity and water absorption. In addition, the leaching results showed that the amounts of heavy metals leached from the crushed bricks were within the regulatory limits. Therefore, substituted bricks have successfully fullfilled the French standards for the suitability of a construction brick.

Due to climate change predictions and catastrophic failure events in recent years, there has been a significant increase of interest concerning the construction of flood leeves. Since it is difficult to obtain suitable natural soils for the construction of leeves as they are limited and non-renewable, research continues into materials that could replace them. To overcome this issue, the possibility of using sediment solidification/stabilization processes has been considered [27]. As a fine, cohesive soil with a low coefficient of permeability, it is possible to use the sediments in the sealing elements of leeves in hydraulic engineering. The main problem with dredged sediments is their high water content, which makes their handling and beneficial use very difficult. An effective method to improve the geotechnical properties and handling of dredged sediments is to prepare (i.e., stabilize) the material using lime or cement-based additives [28,29]. The stabilization and subsequent solidification of the sediment results in a product with a lower moisture content; a stronger internal matrix; and in some cases, a lower potential to leach contaminants.

The main objectives of the current study are a detailed analysis of sediments from the Drava River and the development of new building materials (e.g., bricks) and fillers for flood leeves using sediments as raw material.

## 2. Materials and Methods

### 2.1. Sampling of Sediments

**Figure 1:**
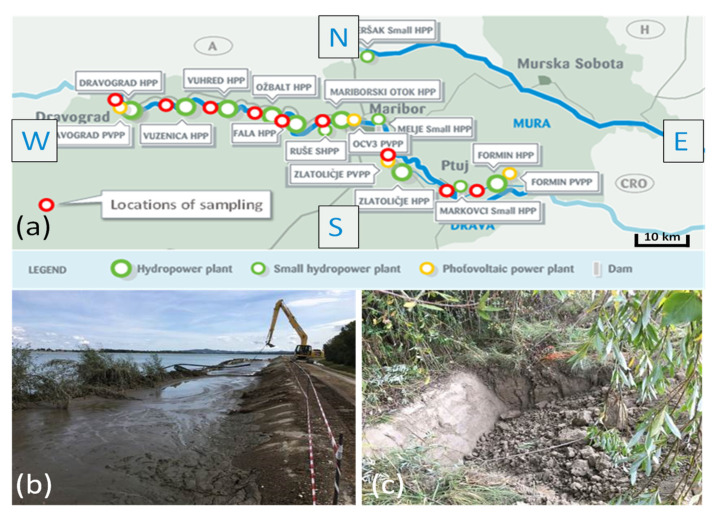
Schematic presentation of the locations (**a**) at which the sediments were sampled with selected sediments on the shore of lake Ptuj—“Lake Ptuj fresh” (**b**); and “Lake Ptuj 9”(**c**).

For the analysis of heavy metals, sediments previously dredged from a depth of 30–50 cm were collected from six sites, namely, the hydroelectric power plants in Dravograd, Vuhred, Ožbalt, Mariborski otok, Melje, and lake Ptuj (see Figure 1 and Table 1). The sediment dredged from lake Ptuj was used for the production of bricks and geotechnical tests. In order to preserve the natural hydration level of the sediment, the samples were stored in a polymer container throughout the entire testing period.

### 2.2. Analysis for Further Use

While all the sediments taken alongside the river and those deposited at the Lake Ptuj have been analyzed, only the samples of sediments “Lake Ptuj 9” and “Lake Ptuj 10” were selected for further investigation regarding their potential use in geotechnics and the clay-based sector due to their availability. Clay from a brick making factory from the eastern part of Slovenia has been selected for the assessment of the suitability of clay replacement by sediment in the brick production industry.

#### 2.2.1. Determining the Contents of Water and Loss on Ignition

The water content was determined by drying the samples in an oven (Kambič, Slovenia). Approximately 1 g of the sample was placed in a ceramic vessel of known mass and heated at 105 °C for 3 h until a constant mass was reached. The water content was calculated by dividing the difference in mass between the initial sample and the dried sample by the mass of the initial sample. The dried sample was then analyzed to determine its mass loss (loss on ignition—LOI) (Eurodent Zlatarna Celje, Slovenia) at 900 °C for 1 h (a heating rate of 10 °C/min), which represents organic matter content, structural water release, and carbonate. Loss on igniton (LOI) was calculated by dividing the mass difference between the fired and dried samples by the mass of the initial sample. The analysis of each sample was performed by averaging over 6 replicates.

#### 2.2.2. Determining the Pb, Zn, and Cd Content

The total contents of the metals Pb, Zn, and Cd were determined by acid leaching. Approximately 0.5 g of the dried sample was placed in a glass beaker, 40 mL of pre-made 1 + 1 (*v*/*v*) nitric(V) acid solution in water (HNO_3_, Honeywell, Fischer, Racine, WI, USA) was added to the sample, and everything was mixed with a glass stirrer. A hot plate was then used to boil the solution until half of the solution had evaporated. The leachate was then filtered through a filter paper into a 50 mL volumetric flask (white label, IDL Gmbh and Co. KG, Nidderau, Germany). Pb, Zn, and Cd contents were determined with a flame AAS by Varian AA240 Atomic Absorption Spectrometer (Varian, Denver, CO, USA) with a multi-element hollow cathode lamp (HCL) Ag/Pb/Zn/Cd (Varian SpectrAA Lamp) using a gas mixture of acetylene/air. The absorption was measured at wavelengths of 217.0 nm, 213.9 nm, and 228.8 nm for Pb, Zn, and Cd, respectively.

#### 2.2.3. Chemical, Mineralogical, and Total Organic Compounds (TOC) Analysis of the Sediment and Clay

The chemical composition of the sediment and clay was determined using a Wavelength Dispersive X-ray Fluorescence (WD XRF) analyzer Thermo Scientific ARL Perform X ((Thermo Fisher Scientific Inc., Ecublens, Switzerland) and using the UniQuant 5.00 software (Thermo Fisher Scientific Inc., Walthem, MA, USA). The LOI of the precursors was determined using the standard method according to EN 196-2. X-ray diffraction in the Bragg–Brentano configuration (XRD) analysis was carried out using a PANalytical Empyrean X-ray diffractometer (Malvern PANalytical Empyrean, Surrey, UK) with CuKα radiation (λ = 1.54 Å) at a voltage of 45 kV and a current of 40 mA, in the 2θ range from 5° to 80° (scan rate = 0.026 °/min). The data were analyzed with the X’Pert High Score Plus diffraction software (PANalytical Empyrean, Surrey, UK) using the PDF 4 + 2015 RDB database for powder diffraction files. Particle size distributions (CILAS 920, Cilas, Orleans Cedex, France) were determined by milling and sieving the examined powders to below 63 µm and then dispersing them with a type C microscan dispersant (Quantachrome Corporation, Boynton Beach, FL, USA). Solid total organic compounds (TOC) were determined by the catalyst-free high temperature combustion system HT 1300, using the direct method following the procedure described in EN 15936:2012: sludge, treated biowaste, soil, and waste—the determination of total organic carbon (TOC) by dry combustion [30] using Multi N/C 3100 (Analytik Jena, Jena, Germany).

#### 2.2.4. Firing Shrinkage, Density, Water Absorption, Bending Strength, and Compressive Strength of Fired Brick-Sediment Samples

Firing shrinkage has been determined by the measurement of dimensions before and after firing on five prisms of dimensions 160 mm × 50 mm × 25 mm. The same prisms have been used for the determination of density, which was performed by the geometrical method by weighing individual samples (Exacta 2200 EB, Tehtnica, Trzin, Slovenia, ±0.01 g) and dividing their weight by their volume. Sample dimensions for shrinkage and density determination were measured using a Vernier Calliper (Mitutoyo, Neuss, Germany) with a precision of ±0.01 mm. Dried prisms were soaked into water for four hours, and water absorption was calculated from differences between dry and wet mass divided by dry mass. Bending strength was performed on the same, dried prisms, while compressive strength was performed on cylinders (radius 50 mm, height 55 mm) using a Toninorm press (Toni Technik, Berlin, Germany, force detection limit 100 N) with a force application rate of 0.05 kN/s.

#### 2.2.5. Leaching Test in Water

The leaching test was performed on samples W-0 (brick-making clay only) and W-8 (50% of brick-making clay, 50% of sediment) according to SIST EN 1744-3:2002 [31]. The clays were crushed and sieved to below 4 mm. The tests were carried out at a liquid-to-solid (L/S) ratio of 10 using a dynamic shaker (ES SM-30, Edmund Buhler GmbH, Bodelshausen, Germany) at a constant speed of 250 rpm for 24 h. The shaker was used for the leaching test. Ultrapure water (0.055 uS/cm) was used as the leaching medium. After 24 h of shaking, the leachates were filtered through a syringe filter and acidified with concentrated HNO_3_. The leachates were analyzed using ICP-MS Agilent Technologies, model 7500 ce&cs (Agilent, Santa Clara, CA, USA).

#### 2.2.6. Weather Resistance and Frost Resistance

The weather resistance of soils was determined as a ration between the uniaxial compressive strength between the saturated and non-saturated samples prepared at the optimal water content. The frost resistance was determined based on the California Bearing Ration (CBR_3_), which included freeze-thaw testing.

## 3. Results and Discussion

### 3.1. Analysis of Potentially Harmful Substances in the Sediments

#### 3.1.1. Water and Organic Matter Content

The water and loss on ignition (LOI) at 950 °C were evaluated in all obtained samples since the potential of the sediments for further processing and possible additional pretreatments depends on them. The results are listed in Table 1. All samples were fairly homogeneous, and the relative standard deviation (RSD) for water content was relatively low, except for Vuhred and Melje samples, where the samples showed an increased heterogeneous character.

The results show that two samples (Ožbalt 1 and Ožbalt 2) contain very high levels of water, which is due to the sediment storage being aligned with water level. The differences between the other samples (storage above water level) are far smaller, with the values of water content varying from 11.5 to 42.1 wt%. The sample from Lake Ptuj 9, which is significantly drier than the other samples, represents the other extreme, with a water content of 11.5 wt%. This is most likely due to the age of this sediment since it had been deposited the longest (i.e., more than 10 years) and therefore had time to dry. No major discrepancies can be seen in the LOI—with the lowest content of 6.2 wt% (Vuhred 2) and the highest of 13.4 wt% (Mariborski otok 1). The LOI of all samples from lake Ptuj are very comparable. The freshest sample (Lake Ptuj fresh) exhibited higher LOI. All samples were homogeneous, and the RSD for the LOI was relatively low, except for the Vuhred 1 and Vuhred 2 samples. The Vuhred location was found to reflect the highest content of a larger particle size, being detected by the naked eye. RSD was increased due to a larger difference in particle size.

#### 3.1.2. Pb, Zn, and Cd in the Lake Ptuj 9 and Lake Ptuj 10 Samples

**Figure 2:**
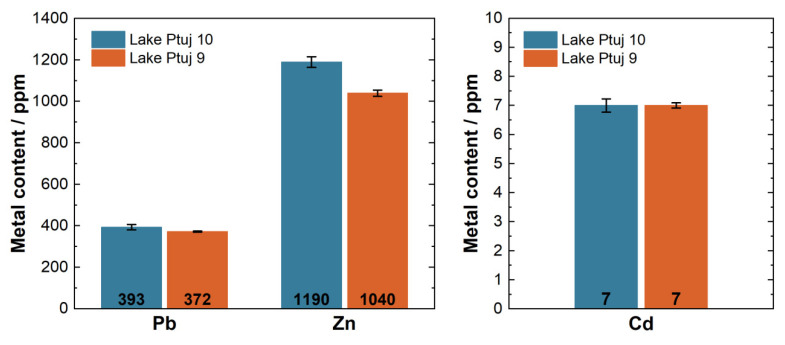
The average total metal contents in the Lake Ptuj 9 and Lake Ptuj 10 samples.

As Pb, Zn, and Cd were recognized in the previous studies listed, the levels thereof were also determined in all the samples. However, the focus was on the samples from Lake Ptuj 9 and Lake Ptuj 10 as these samples would be analyzed and tested for further use in the building sector. The sediments from more than 20 sites were sampled in the River Drava, and there were no significant differences in heavy metal concentrations. The Ptuj site was chosen because it has the largest amount of sediment that could be used for further applications, and due to logistical reasons as these two locations are the easiest to access with heavy machinery, with all the needed transportation routes. The performed analysis confirmed that these two samples did not significantly vary from the others in terms of heavy metal content. The average levels determined for the three metals of interest are shown in Figure 2.

The sediments were found to exceed the allowed levels for unrestricted transportation and deposition for all of the three metals of interest. Zn contamination reached up to 1190 (±0.01) ppm. The Pb content was 393 ppm in the Lake Ptuj 10 sample and 372 ppm in the Lake Ptuj 9 sample. Across six replicates, the RSD of Pb in both samples was 3%. Cd was found in relatively low amounts, as expected, but still exceeded the legal values [32]. The Cd content was determined to be 7.1 ppm in both samples, with RSD values of 6.6% and 3.3% in the samples from Lake Ptuj 9 and Lake Ptuj 10, respectively.

According to the decree on the burdening of soil with waste spreading—the decree on soil pollution [33], the concentrations of Pb, Zn, and Cd also exceeded the limits for potential backfilling on agricultural land.

**Figure 3:**
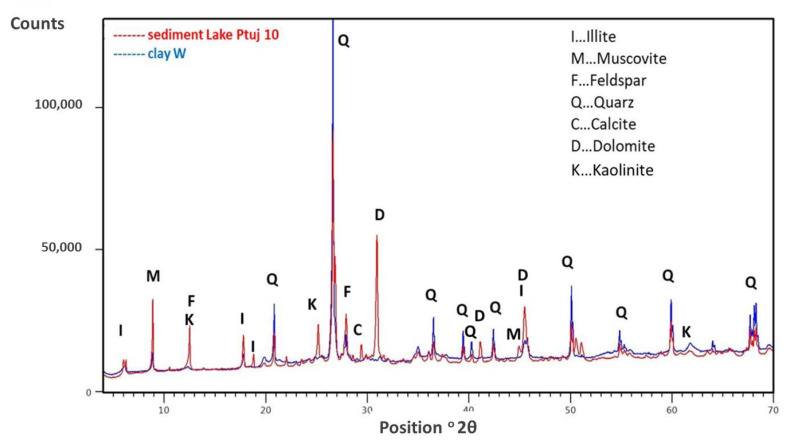
XRD diffractograms—mineralogical compositions of the Lake Ptuj 10 sediment and the clay W.

These results led to further work addressed at possibly using these sediments in the building sector. For this purpose, the chemical and mineralogical compositions of deposit of sediment from Lake Ptuj were determined and are listed in Table 2 and given in Figure 3.

### 3.2. Evaluating the Potential Use of Sediments for the Production of Clay Bricks

When evaluating specific secondary raw materials in the clay-based sector, it is also important to determine the mineralogical and chemical composition of the materials, in addition to an environmental assessment, to verify whether the type of additive will replace the clay or sand content of the virgin raw material.

In general, the suitability of the raw material or additives for the production of brick products is thus verified on following parameters [20]:

- The clay content,
- The free silica and carbonate content,
- The particle size distribution,
- The moisture content per wet mass at moulding (plasticity),
- The shrinkage on drying, and
- The properties after firing.

The mineralogical composition of the clays or sediments is an important factor in the identification of the raw material or potential additives in the clay brick production process. Additionally, the properties of fired samples depend considerably on the minerals present in the raw materials. The content of clay particles in brickmaking clay can range from 20 to 60%; in practice it is usually 30%. The content of quartz is also an important parameter because silica affects both the properties of the product and the drying process (reducing sensitivity), as well as the firing process, where the phase transformation of silica at 573 °C in the cooling phase must be taken into account since it is associated with volume changes and can lead to cracking of the products if the cooling process is not properly managed. The carbonate content, if finely dispersed, can be as high as 20–25%; only lime inclusions larger than 1 mm can be a problem. Higher carbonate content may cause the precipitation of water-soluble salts after firing; while this does not compromise the quality of the product, it may be aesthetically disturbing. The particle size distribution of the raw material also has a significant effect on the process ad properties; it indirectly reflects the content of clay minerals because, based on the Winklers diagram, particles smaller then 2 µm are ascribed to the clay minerals, while particles bigger then 20 µm represent quartz [34]. The plasticity of the clay is important in several respects; if the raw material is not sufficiently plastic, it is difficult to shape, the dry samples are brittle, and damage occurs during normal transport, while if the clay is too plastic, it dries slowly and retains moisture inside, which can lead to the cracking or warping of the samples. A high moisture content per wet mass is also not desirable because more energy has to be used in the drying process to remove the moisture. The normal moisture content per wet weight of brick clays is between 17 and 25%.

In general, the chemical (see Table 2) and mineralogical composition (Figure 3) of sediments is similar to the mineralogical composition of clay W except that sediment contains feldspar, calcite, and dolomite, and less quartz then clay. In the present case it can be noticed that both clay and sediment contain a certain amount of organic component (with TOC being 1.8 and 3.4% for clay W and sediment, respectively). In clay materials, in general, decomposition up to 500 °C presents organic matter decomposition and the release of constitutional water [35]. The decomposition at 550 °C (LOI at 550 °C) covers TOC and structural water release, while the LOI at 950 °C presents, beside those two, the decomposition of carbonates, and in present case the LOI at 550 °C amounts to 9.1 and 7.2%, while the LOI at 950 °C amounts to 10.9 and 15.8%, for clay and sediment, respectively. The carbonates in the sediment (calcite, dolomite) were also confirmed by XRD (Figure 3) with corresponding CaO and Mgo in the chemical analysis of the sediment (Table 2).

**Figure 4:**
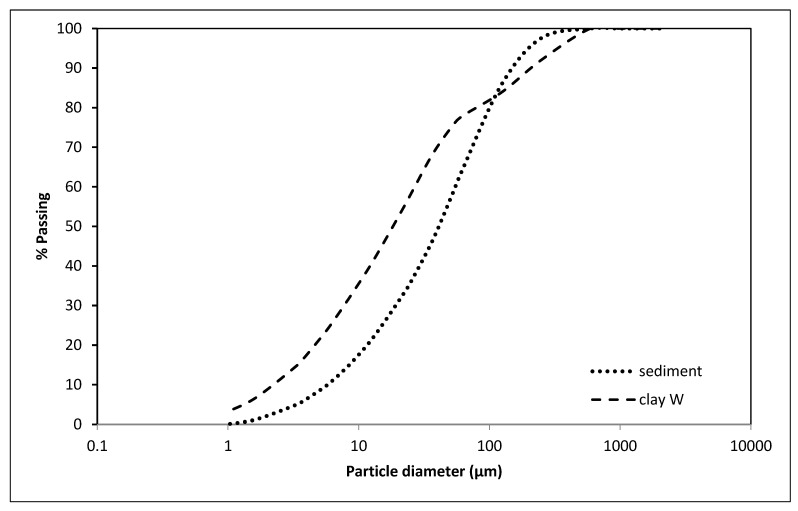
Particle size distribution of the W clay and sediment from Lake Ptuj 10.

Based on the chemical and mineralogical parameters and particle size distribution (Figure 4), sediment has been assessed as suitable for use in the clay-based sector [20,34], and further analysis of clay mixtures containing sediment have been performed.

**Figure 5:**
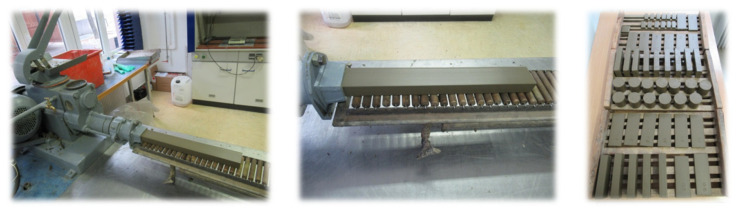
Sample preparation with a de-airing extruder.

The particle size of the sediment Lake Ptuj 10 was below 200 µm, with a similar distribution to the brick-making clay (Figure 4). Based on these analyses, a large amount of brick-making clay could be replaced by such a sediment, and therefore mixtures with a substitution of up to 50 wt% sediment were prepared. The samples with dimensions of 150 × 50 × 25 mm^3^ were extruded in a vacuum de-airing extruder (Figure 5) and fired at 950 °C for 2 h by applying a heating rate of 10 °C/min.

The shrinkage following extrusion drying was measured after extrusion, and the firing shrinkage, water absorption, density, bending strength, and compressive strength were assessed after firing. The results are shown in Table 3.

**Figure 6:**
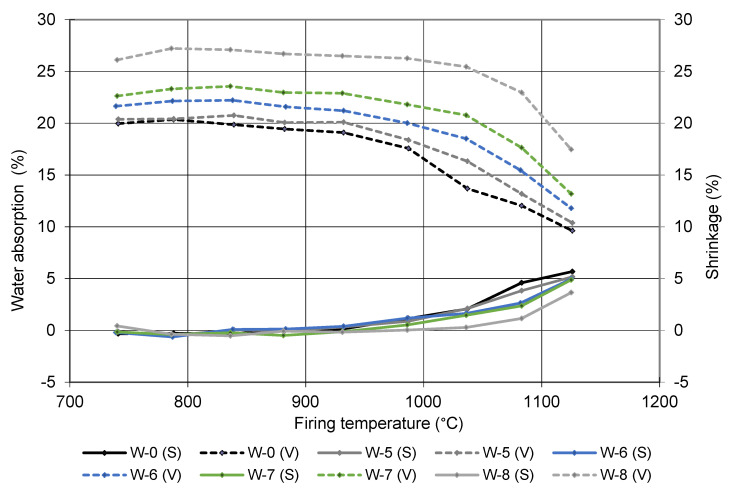
Shrinkage (S) and water absorption (V) independence of firing temperature.

Sample W-0 contains only brick-making clay, while samples W-5, W-6, W-7, and W-8 contain 10, 20, 30, and 50 wt% of sediment (the rest is brick-making clay). The most significant effect of adding sediment to the brick-making clay is a more porous structure, which translates into higher water absorption and consequently lower mechanical strengths. The water absorption of the pure brick-making clay (sample W0) is 17.2%, with a corresponding compressive strength of 33 MPa, while sample W8, in which half of the clay was replaced by sediment, reached 25 MPa with 25.1% water absorption. The addition of sediment also increases the shrinkage after drying and decreases the shrinkage after firing. The same trend is also evident from the gradient firing results presented in Figure 6, where the water absorption and shrinkage increase towards the firing temperature. The analysis of the firing in the gradient kiln gives information on the linear shrinkage and water absorption as a function of firing temperature and provides information about sensitivity of clay to the deformation over a certain firing temperature range. All of the pressing temperatures indicate that the water absorption increases significantly with the addition of sediment. On the one hand, this can be attributed to the higher TOC and LOI (Table 2), which means that there are more organic compounds in the sediment that burn off and leave voids. On the other hand, the sediment contains more carbonates (dolomite, calcite in Figure 3), which also decompose below the firing temperature of clay bricks, leaving voids in the structure.

**Figure 7:**
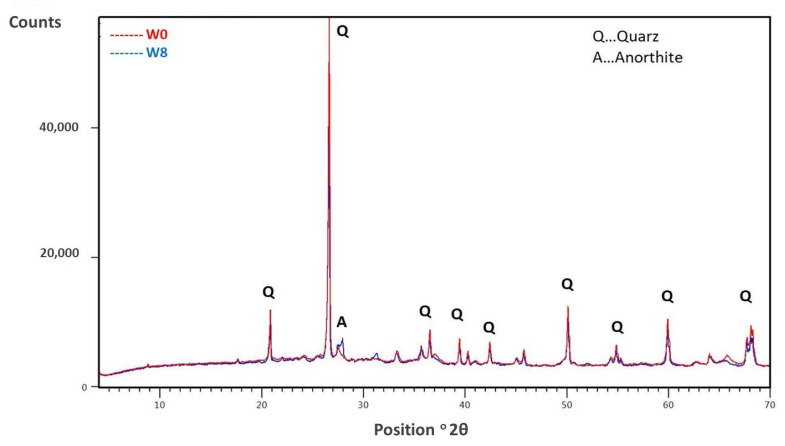
XRD pattern of sample W0 fired at 950 °C and sample W8 (containing 50% sediment) fired at 950 °C.

Comparing the XDR diffractograms of W0 and W8 after firing at 950 °C in Figure 7, we find that there are no major differences in mineralogical composition; mainly quartz is present. Based on the chemical compositions (Table 2), it appears that additional peaks (at 28°), which start to emerge in W8, could correspond to anorthite [36].

In addition, in selected samples (W-0 and W-8, the first being clay only without sediment addition, the second with the maximum addition), the levels of Pb, Zn, and Cd in the leachate were also determined by ICP-MS. The metal contents in the leachate of each specimen are shown in Table 4. This shows that the production process has successfully immobilized the heavy metals and that the leached concentrations are in compliance with the current regulations permitted by the (decree on waste).

### 3.3. Evaluating the Potential Use of Sediment for Geotechnical Purposes

#### 3.3.1. Geomechanical Properties of the Sediment

River sediments are currently pumped from the bottom of lake Ptuj and deposited on its shores, where they are subjected to a natural drying process (Figure 1). Since a large amount of this semi-dry material is already present, new possibilities for its use need to be explored. In addition, environmental legislation in this area is expected to tighten in the coming years, which means that the possible use of this material in geotechnical construction should be investigated. For geomechanical testing sediment, Lake Ptuj 10 was used. Due to the high-water content of the naturally dried sediment (over 50 wt%), the geomechanical properties are very poor (see Table 5), so the dehydrated sediment alone could not be used for geotechnical structures such as road construction, levees, or even backfill layers.

It should be emphasized that in geotechnical engineering, the term water content is used for the ratio between the weight of water and the weight of dry material in the sample. The usability of the sediment could be achieved by mixing the sediment with inorganic binders such as quicklime [46,47].

#### 3.3.2. Geomechanical Properties of the Sediment/Quicklime Mixture

The geotechnical laboratory studies of the potential use of the sediment were based on the preparation of a mixture that contained the optimum amount of binder. Quicklime was used as a binder because of its ability to bind surplus water from the sediment well. Since the natural sediment had a different water content, three main groups were proposed, each with an average water content of 33 wt%, 43 wt%, or 53 wt%, respectively (intervals ±5 wt%, see Table 6). The groups reflect natural conditions where the water content is lower in the upper layers. For each group, the optimum amount of quicklime was determined and then the optimum parameters for installation were determined. Several geotechnical tests were performed in the laboratory on the optimum mixes, prepared according to the installation parameters. The main criterion in the selection of the optimum mixes was the determination of the minimum amount of quicklime that would allow the mixes to be geotechnically workable while providing sufficient compressive strength for the layers of the levees.

The parameters for installation, i.e., optimum water content and maximum dry density, were determined using standard Proctor tests. Since the reaction between quicklime and the water in the sediment is intense, the samples were prepared so that the sediment was mixed with the right amount of quicklime and then allowed to stand for 24 h before compacting. Geomechanical tests were then performed in the laboratory to determine the unconfined compressive strength, the shear strength, and the water permeability; the weather and frost resistance; and the stiffness of the compacted material (oedometer tests).

**Figure 8:**
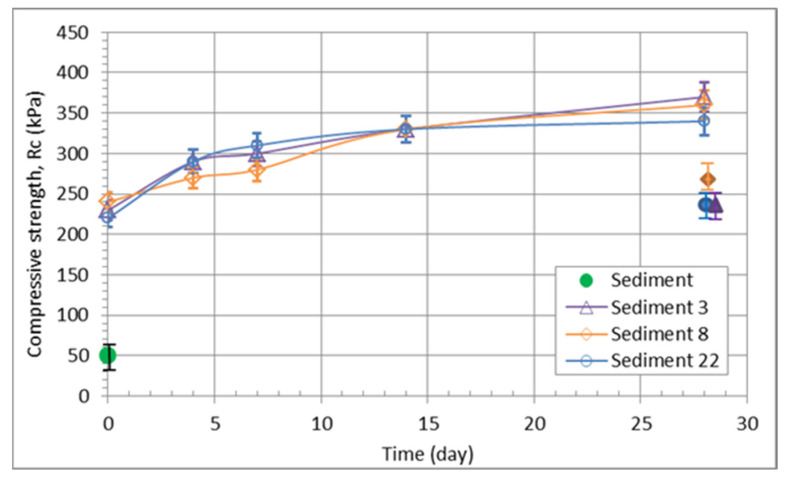
Unconfined compressive strength of the various mixtures; sediments only and sediments containing 3, 8, and 22% of lime additives (full markers representing the fully saturated samples, except the green one).

The results of unconfined compressive strength show that the sediment with quicklime reaches values up to five times higher than those of the solid sediment (Figure 8). The values also increase slightly up to 14 days after the compaction process. However, it is interesting to note that the amount of quicklime added has no effect on the compressive strength of the mixes (Figure 7). After 28 days, the fully saturated samples were also tested, and the values obtained were about 40% lower than those determined on the unsaturated samples (see Table 7). The uniaxial compressive strength could be increased by adding a few percent of cement in the mixture [48] or fly ash [49,50]. According to TSPI 05.200. [51], the tested materials have middle to high frost resistance and high whether resistance (R > 0.7). The oedometer modulus for the samples with quick lime content increased four times in comparison with the sediment, but the difference between 3% and 22% of adding quick lime is only 2.4 MPa.

**Figure 9:**
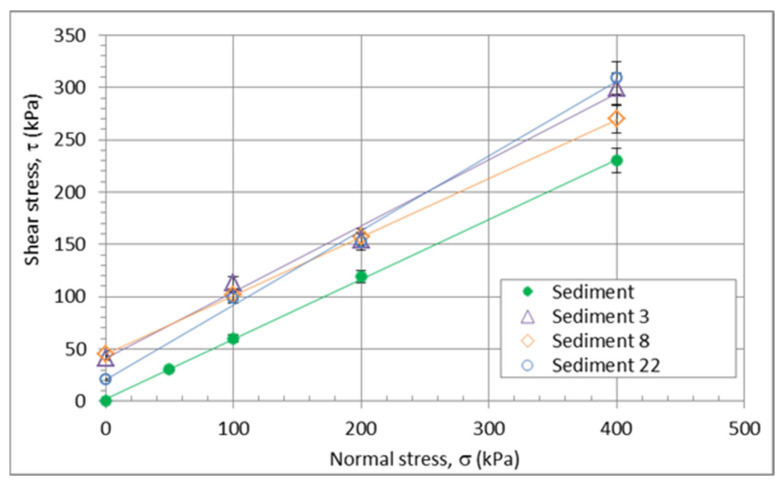
Shear strength of the various mixtures; sediments only and sediments containing 3, 8, and 22% of lime additives.

A necessary parameter for levees is the shear strength of the material. For this reason, direct shear tests were performed under water. Neither the amount of quicklime in the mixtures nor the age of the mixtures had any effect on the shear parameters (Figure 9). The only difference from the natural sediment is a higher initial strength—cohesion. The results of the shear tests show that it is possible to use it for construction purposes. The main criterion in the selection of the optimum mixes was the determination of the minimum amount of quicklime that would allow the mixes to be geotechnically workable while providing sufficient compressive strength for the layers of the levees with a slope of 2:3 (height:length), which is comparable to what is possible with natural gravel or sandy gravel soils. When the levees are used for flood protection, the permeability of the material plays an important role.

**Figure 10:**
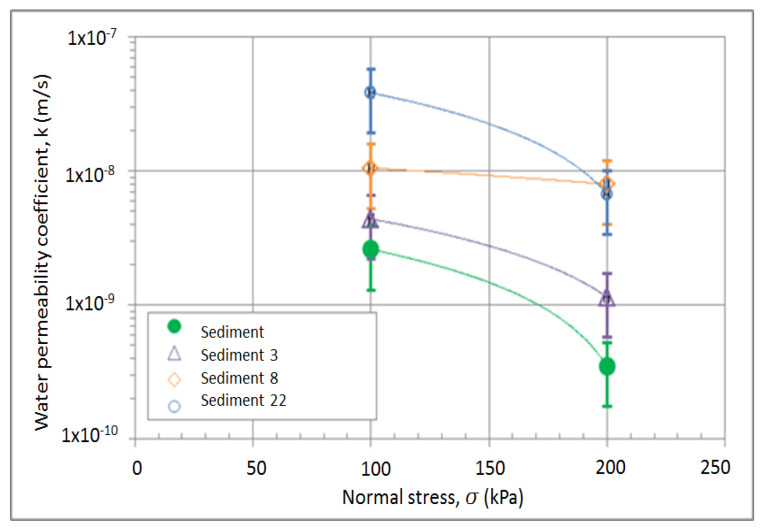
Results of water permeability coefficient of the mixtures; sediments only and sediments containing 3, 8, and 22% of lime additives.

The coefficient of water permeability is independent of the amount of quicklime as well as of the time after compaction, but it is slightly dependent on the load. The only parameter that has an influence on the water permeability coefficient is the particle size distribution of the sediment. However, the values range between 10^−8^ and 10^−9^ m/s, which classifies the mixtures as low permeability material such as silty clay soils (Figure 10).

Based on all the obtained results, we conclude that the mixtures consisting of sediments from the area of lake Ptuj and quicklime as a binder can be used for the following purposes: (i) the construction of levees and backfill layers, (ii) the construction of watertight layers; and (iii) as a backfilling material for mine sinkholes or other degraded areas.

## 4. Conclusions

In the first phase, numerous sediment samples were collected from the reservoir and various locations along the Drava River and analyzed for the presence of heavy metals. Total concentrations of heavy metals were determined by acid digestion and measured using AAS. The measurements confirmed elevated levels of Zn, Pb, and Cd, with Zn content being the highest. The heavy metal concentrations determined still allow for the use of these sediments for the production of construction materials without the need for pretreatment.

Ther chemical and mineralogical analysis of the sediment have shown some differences in the compositions; clay exhibits a higher amount of silica than sediment, while sediment contains more carbonates and feldspar. The particle size of the sediment was below 200 µm, which classifies the material as suitable for further use in certain areas of the building sector. Based on these analyses, the potential use of dredged sediment was assessed by replacing natural brick-making clay with sediments by adding it in proportions of 10, 20, 30, and 50 wt% and fired at a temperature of 950 °C. The addition of sediment slightly increased the drying shrinkage, from 8.5% for the clay bricks to 9.2% for the bricks containing 30% sediment. However, the addition of sediment decreased the shrinkage after firing from 1.3 to 0.4% for the control and the bricks with sediment, respectively, and also increased the water absorption of the fired products. While the mechanical properties of bricks with the addition of 10 or 20 wt% sediment remained unchanged, the compressive strength decreased from 33 to 28% when the addition of sediment was increased to 30 wt%. The leachates of the bricks produced with the sediments were analyzed for the concentration of heavy metals, specifically for the Pb, Zn, and Cd, which have been already in the past recognized as potential pollutants present in river Drava. The analysis showed that the production process successfully immobilized the heavy metals and that the leached concentrations were in compliance with current regulations.

The natural moisture content of the one-year-old naturally deposited sediment dredged from the river was too high to be used as backfill material alone, requiring the addition of binders to dry it. The optimum moisture content of the sediment in the mixture was reduced to a value at which it was possible to achieve compaction on the construction site while maintaining adequate unconfined compressive strength and shear resistance. Using suitable machinery, the quicklime could be added to the sediment in two different ways: (i) treatment (mixing quicklime in the sediment) performed on the levees built by Lake Ptuj to a depth of 0.3–0.5 m; and (ii) treatment (mixing quicklime in the sediment) performed at the installation site, in a layer of sediment prepared 0.3–0.5 m thick. At least 24 h after mixing the sediment with quicklime, the mixture was then compacted.

It has been proved that such sediments can be used for both of the investigated final uses: as partial replacement in clay brick production and for soil stabilization purposes. Since sedimentation in front of dams of hydropower plants presents one of the major problems for hydropower plant management worldwide, the presented methodology might help one to also solve this issue for other types of sediments. Thus, following one of the paradigms of the circular economy, where waste from one sector is used as a raw material in another, and contributing to environmental protection and sustainable development, the potentially beneficial use of river sediments is hereby confirmed.

## Figures and Tables

**Table 1:** Water ^1^ and LOI at 950 °C ^2^ levels in the tested Drava River sediments.

Sample	Water (%)	RSD Water (%)	LOI at 950 °C (wt%)	RSD LOI (%)
Dravograd 1	29.2	3.3	12.3	2.8
Dravograd 2	29.3	1.7	12.2	1.9
Vuhred 1	27.2	8.1	10.8	13.0
Vuhred 2	21.8	13.8	6.2	16.8
Melje 1	56.9	4.6	6.8	2.9
Melje 2	30.9	13.9	7.9	9.8
Čolnarna 1	24.8	2.7	9.3	5.5
Čolnarna 2	23.8	5.4	8.3	3.0
Mariborski otok 1	27.6	1.9	13.4	1.3
Mariborski otok 2	28.2	1.0	11.2	0.7
Ožbalt 1	67.1	0.4	7.0	7.6
Ožbalt 2	71.3	0.6	7.0	1.8
Lake Ptuj fresh	42.1	1.9	8.5	0.4
Lake Ptuj 1	35.5	0.5	10.0	2.1
Lake Ptuj 2	29.8	5.8	10.8	2.2
Lake Ptuj 3	32.9	1.1	10.7	0.8
Lake Ptuj 4	27.7	2.2	10.8	2.1
Lake Ptuj 5	33.5	5.5	10.4	2.2
Lake Ptuj 6	30.8	3.0	11.2	0.9
Lake Ptuj 7	30.5	9.5	11.1	1.1
Lake Ptuj 8	32.9	5.9	10.8	2.9
Lake Ptuj 9	11.5	3.8	12.4	1.4
Lake Ptuj 10	30.4	1.7	10.7	1.3

^1^ Water content is calculated as the ratio between the weight of water and the initial sample weight. ^2^ LOI is calculated as the ratio between weight of the fired sample and the initial dry sample weight.

**Table 2:** Chemical compositions, TOC, and LOI at 550 and 950 °C of the Lake Ptuj 10 sediment and the W clay used for bricks (in wt%).

	W0	Lake Ptuj 10
Na_2_O	0.85	1.46
MgO	1.21	6.33
Al_2_O_3_	20.22	18.74
SiO_2_	66.30	53.28
P_2_O_5_	0.09	0.28
SO_3_	0.00	0.58
K_2_O	2.14	2.73
CaO	0.54	8.46
TiO_2_	1.22	0.94
V_2_O_5_	0.03	0.03
Cr_2_O_3_	0.01	0.02
MnO	0.10	0.10
Fe_2_O_3_	6.93	6.52
ZnO	0.01	0.19
As_2_O_3_	0.10	0.11
Rb_2_O	0.02	0.02
SrO	0.01	0.03
ZrO_2_	0.07	0.04
others	0.22	0.18
TOC	1.8	3.4
LOI at 550 °C	9.1	7.2
LOI at 950 °C	10.9	15.8

**Table 3:** Properties of the dried and fired samples.

Designation	Addition of Sediment from Lake Ptuj 10 (wt%)	Shrinkage after Drying (%)	Shrinkage after Firing (%)	Density (g/cm³)	Water Absorption (%)	Bending Strength (MPa)	Compressive Strength (MPa)
W-0	0	8.5	1.3	1.76	17.2	11.2	33
W-5	10	8.9	1.1	1.73	18.1	13.7	31
W-6	20	9.2	0.6	1.69	19.9	13.6	35
W-7	30	9.2	0.4	1.66	21.4	10.7	32
W-8	50	9.2	0.0	1.52	25.1	7.1	25

**Table 4:** Metal content levels in the leachates of samples W-0 and W-8 with the legal limit values (decree on waste).

	Zn (ppb)	Cd (ppb)	Pb (ppb)
W-0	0.52	0.04	<0.01
W-8	1.27	0.03	0.35
legal limit	35.0	0.25	3.5

**Table 5:** Physical and mechanical properties of the naturally dried sediment.

Property	Standard	Value
Initial Moisture Content (w) (wt%) ^1^	SIST EN ISO 17892-1 [37]	42–60
Specific Gravity (γs) (Mg/m^3^)	SIST EN ISO 17892-3 [38]	2.69–2.70
Liquid Limit (w_L_) (%)	SIST EN ISO 17892-12 [39]	49.1–69.0
Plastic Limit (w_P_) (%)	SIST EN ISO 17892-12 [39]	41.9–49.0
Consistency Index (Ic) (-)	SIST EN ISO 17892-12 [39]	0.4–1.6
Particle Size Distribution:		
Particle (<2.0 mm) (%)	SIST EN ISO 17892-4 [40]	90–100
Particle (<0.063 mm) (%)	SIST EN ISO 17892-4 [40]	53.9–61.4
Particle (<0.002 mm) (%)	SIST EN ISO 17892-4 [40]	3.8–4.4
Classification	SIST EN ISO 14688-2 [41]	mSi–hSi
Optimum Water Content—Standard Proctor Test (w_opt_) (%)	SIST EN 13286-2 [42]	29.5
Maximum Dry Density—Standard Proctor Test (ρ_d,max_) (Mg/m^3^)	SIST EN 13286-2 [42]	1.33
Unconfined Composite Strength After Compaction (q_u_) (MPa)	SIST EN ISO 17892-7 [43]	0.05
Eodometer Modulus (MPa)	SIST EN ISO 17982-5 [44]	2.55
Shear Resistance:		
Friction Angle (f’) (°)	SIST EN ISO 17892-10 [45]	30.5
Cohesion (c’) (kPa)	SIST EN ISO 17892-10 [46]	0.6

^1^ Initial moisture content is calculated as the ratio between the weight of water and weight of dry sample.

**Table 6:** Composition of the optimal sediment/quicklime mixtures and their installation parameters.

Mixture Designation	Average Moisture Content of the Sediment w, (wt%)	Quicklime Content (wt%) *	Optimal Water Content Standard Proctor Test w_opt_ (wt%)	Maximum Dry Density—Standard Proctor Test ρ_d,max_ (mg/m^3^)
Sediment	60	0	29.5	1.33
Sediment 3	33	3	27.1	1.42
Sediment 8	43	8	24.4	1.52
Sediment 22	53	22	26.3	1.43

* The percentage of quicklime in the mixture means the mass of the quicklime added to the dry mass of the sediment.

**Table 7:** Results of the laboratory-based geotechnical tests for sediment only and sediments containing 3, 8, and 22% of lime additives.

Mixture Designation	Unconfined Compressive Strength, Rc (MPa)						Shear Strength		Weather Resistance	Frost Resistance	Oedometer Modulus
	0 days	After 4 days	After 7 days	After 14 days	After 28 days	Saturated, 28 days	Friction angle, f’ (°)	Cohesion, c’ (kPa)	R (-)	-	E_oed,200 kPa_ (MPa)
Sediment	0.05						30.5	0.6	-	-	2.55
Sediment 3	0.23	0.29	0.30	0.33	0.37	0.21	32.5	41	0.86	middle to high	11.2
Sediment 8	0.24	0.27	0.28	0.33	0.36	0.24	29.0	45	0.85	middle to high	12.5
Sediment 22	0.22	0.29	0.31	0.33	0.34	0.21	35.5	21	0.78	middle to high	13.6

## Data Availability

Not applicable.
